# Progress and trends in myocardial infarction-related long non-coding RNAs: a bibliometric analysis

**DOI:** 10.3389/fmolb.2024.1382772

**Published:** 2024-07-29

**Authors:** Qingkun Meng, Hao Tan, Chengfu Wang, Zhijun Sun

**Affiliations:** ^1^ Department of Cardiology, The People’s Hospital of China Medical University, Shenyang, China; ^2^ Department of Thoracic and Esophageal Radiotherapy, Liaoning Cancer Hospital and Institute, Shenyang, China; ^3^ Department of Cardiology, Shengjing Hospital of China Medical University, Shenyang, China

**Keywords:** myocardial infarction (MI), long non-coding RNAs (lncRNAs), bibliometric, web of science core collection, cluster analysis

## Abstract

**Background:**

Myocardial infarction (MI), a critical condition, substantially affects patient outcomes and mortality rates. Long non-coding RNAs (lncRNAs) play a critical role in the onset and progression of MI. This study aimed to explore the related research on MI-related lncRNAs from a bibliometric perspective, providing new clues and directions for researchers in the field.

**Methods:**

A comprehensive search was conducted on 7 August 2023, using the Web of Science Core Collection (WoSCC) database to compile a dataset of all English-language scientific journals. The search gathered all relevant publications from January 2000 to August 2023 that pertain to MI-related lncRNAs. Data on countries, institutions, journals, authors, and keywords were collected, sorted, statistically analyzed, and visualized using CiteSpace 6.2.R4, VOSviewer 1.6.19, an online bibliometric analysis platform (http://bibliometric.com), and the bibliometric package in R-Studio 4.3.1. Articles were screened by two independent reviewers

**Results:**

Between January 2000 and August 2023, a total of 1,452 papers were published in the research field of MI-related lncRNAs. The year with the most publications was 2020, accounting for 256 papers. The publication volume displayed an exponential growth trend, fitting the equation y = 2.0215e^0.2786x^, R^^2^ = 0.97. In this domain, China leads in both the number of published papers (N = 1,034) and total citations, followed by the United States, Germany, Iran, and Italy. The most productive institution is Harbin Medical University (N = 144). The European Review for Medical and Pharmacological Sciences had the highest number of publications (N = 46), while Circulation Research had the most citations (TC = 4,537), indicating its irreplaceable standing in this field. Research mainly focuses on the cardiovascular system, cellular biology, physiology, etc. The most productive author is Zhang Y. Apart from “Myocardial Infarction” and “LncRNA,” the most frequent keywords include “expression,” “atherosclerosis,” and “apoptosis.” Cluster analysis suggests current research themes concentrate on cardiovascular diseases and gene expression, cardiac ischemia/reperfusion injury and protection, expression and proliferation, atherosclerosis and inflammatory response, among others. Keyword bursts indicate recent hot topics as targeting, autophagy, etc.

**Conclusion:**

This bibliometric analysis reveals that research on MI-related lncRNAs has rapidly expanded between January 2000 and August 2023, primarily led by China and the United States. Our study highlights the significant biological roles of lncRNAs in the pathogenesis and progression of MI, including their involvement in gene expression regulation, atherosclerosis development, and apoptosis. These findings underscore the potential of lncRNAs as therapeutic targets and biomarkers for MI. Additionally, our study provides insights into the features and quality of related publications, as well as the future directions in this research field. There is a long road ahead, highlighting the urgent need for enhanced global academic exchange.

## 1 Introduction

Myocardial infarction (MI), a severe form of coronary heart disease (CHD), results from prolonged ischemia and hypoxia due to blocked coronary arteries, posing significant life-threatening risks. According to the World Health Organization (WHO), cardiovascular diseases remain the global leading cause of death, with CHD accounting for the largest proportion. According to statistics, approximately one million individuals in China experience acute myocardial infarctions annually, and the incidence rate is gradually increasing (Author Anonymous, 2021). Therefore, identifying new therapeutic targets for myocardial infarction is of paramount importance. Long non-coding RNAs (lncRNAs) are a class of non-coding RNA molecules longer than 200 nucleotides that do not encode proteins. These lncRNAs are widely implicated in vital cellular processes such as metabolism, signal transduction, proliferation, differentiation, apoptosis, and cell death, and are particularly relevant to the onset and progression of cardiovascular diseases (Jiao et al., 2019). In recent years, there has been a continuous increase in the number of publications related to lncRNAs, which remain a focal point of RNA functional studies.

Bibliometrics, an interdisciplinary field utilizing mathematical and statistical methods, effectively quantifies vast bodies of knowledge across various domains. This methodology excels in mapping research trends, analyzing contributions by countries, institutions, and authors, and identifying emerging topics within a scientific discipline. Unlike systematic reviews and meta-analyses, which synthesize findings from select studies to assess specific hypotheses, bibliometrics offers a broader overview, providing visualizations of data trends and network relationships. These capabilities make it particularly valuable for exploring extensive datasets and forecasting future research directions, thereby supporting the development of therapeutic policies and clinical guidelines. This study leverages bibliometric tools to delve into myocardial infarction-related lncRNA research, uncovering new insights and potential areas for further investigation.

## 2 Methods

### 2.1 Data acquisition and search strategy

**FIGURE 1:**
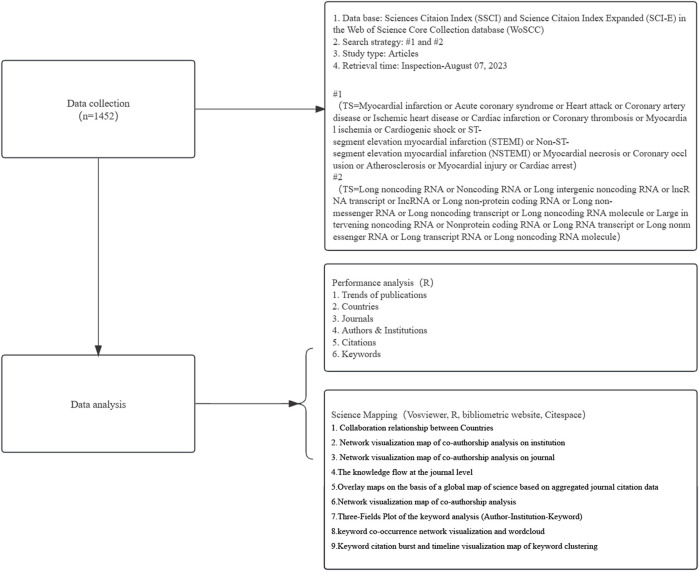
Flowchart of data collection and study design. This figure outlines the step-by-step process used in our study to collect and analyze data on MI-related lncRNAs. The process begins with a comprehensive search of relevant database Web of Science, using specific keywords. Articles were then screened based on predefined inclusion and exclusion criteria. Eligible studies were subjected to data extraction and quality assessment. The extracted data were analyzed using bibliometric methods to identify trends, key research areas, and influential publications in the field of MI-related lncRNAs. Additionally, the figure includes notes on the criteria used for study selection and the statistical methods applied for data analysis.

This study encompasses all English-language publications and reviews on MI-related lncRNAs over the past 23 years (from 1 January 2000, to 7 August 2023) in the Web of Science Core Collection (WoSCC). WoSCC is recognized as one of the most comprehensive and authoritative platforms for accessing global academic information, containing over 12,000 journals. It serves as a typical citation database that includes literature abstracts, citation data, and research collaboration information, making it widely used for bibliometric analysis. The database provides direct support for exporting reference files in specific formats required by bibliometric software. To ensure consistency and reduce bias due to database updates, all literature for this study was retrieved and downloaded from the Science Citation Index Expanded of WoSCC on 7 August 2023. Search terms were based on the Medical Subject Headings (MeSH) terms and relevant publications on heart failure and depression, including: (“Myocardial infarction” or “Acute coronary syndrome” or “Heart attack” or “Coronary artery disease” or “Ischemic heart disease” or “Cardiac infarction” or “Coronary thrombosis” or “Myocardial ischemia” or “Cardiogenic shock” or “ST-segment elevation myocardial infarction” (STEMI) or “Non-ST-segment elevation myocardial infarction” (NSTEMI) or “Myocardial necrosis” or “Coronary occlusion” or “Atherosclerosis” or “Myocardial injury” or “Cardiac arrest”) and (“Long noncoding RNA” or “Noncoding RNA” or “Long intergenic noncoding RNA” or “lncRNA transcript” or “lncRNA” or “Long non-protein coding RNA” or “Long non-messenger RNA” or “Long noncoding transcript” or “Long noncoding RNA molecule” or “Large intervening noncoding RNA” or “Nonprotein coding RNA” or “Long RNA transcript” or “Long nonmessenger RNA” or “Long transcript RNA” or “Long noncoding RNA molecule”). The complete bibliographic characteristics and citation information were independently retrieved and downloaded by two researchers (Qingkun Meng and Hao Tan) on the same day (7 August 2023) from publicly available databases. This study did not involve animal experiments and did not require ethical approval. Figure 1 shows the flowchart of the data collection process.

### 2.2 Methods and tools for bibliometric and knowledge graph analysis

R-Studio 4.3.1 software’s bibliometric package and the online bibliometric analysis platform (http://bibliometre.com/) (Aria and Cuccurullo, 2017) were used to map collaboration networks among research entities across the world. The data clustering of authors, journals, institutions, countries, and keywords involved in this study was organized, statistically analyzed, and visualized in a composite network using CiteSpace 6.2.4.R4, VOSviewer 1.6.19, and collaborative bibliometric packages.

VOSviewer software was used to visualize the co-authorship analyses of institutions and authors with a document threshold of 15, as well as the co-occurrence analysis of keywords with an occurrence threshold of 5. CiteSpace was mainly used for reference co-citation analysis, keyword burst detection, and drawing timeline views of reference clusters. In the visual graphs, the colors of the nodes and connections represent the period in which the articles were published. The overall time span from 2000 to 2023, with 1 year per slice, was divided into 15 different time slices corresponding to 15 different colors.

## 3 Results

### 3.1 General information on publications and annual publication trends

Between January 2000 and August 2023, a total of 1,452 papers were published. The year with the highest number of publications was 2020, accounting for 256 papers, while no papers were published in 2004 and 2006. The annual growth rate was 15.41%. The publication trend showed fluctuating growth and can be divided into three phases:

First Phase: From 2000 to 2009, the number of published papers was relatively low, averaging around 3.6 papers per year, with a cumulative total of 23 papers.

Second Phase: From 2010 to 2017, the number of published papers significantly increased, averaging about 37.5 papers per year, with a cumulative total of 273 papers.

Third Phase: From 2018 to August 2023, the number of published papers reached its peak, averaging around 177.7 papers per year, with a cumulative total of 1,152 papers.

**FIGURE 2:**
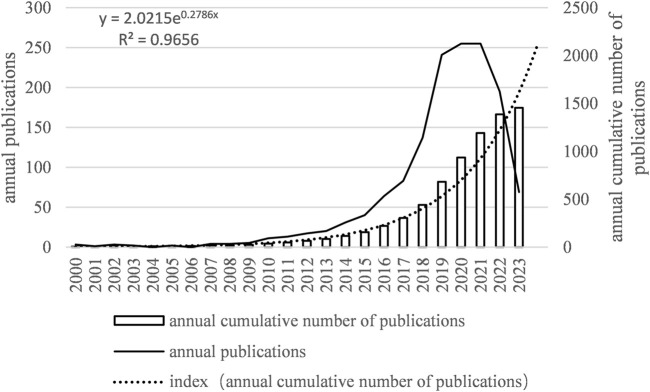
Number of annual articles on MI-related lncRNAs. The bar graph shows the number of publications per year. The line graph overlays the total number of citations received each year. Notably, there is a sharp rise in both publications and citations in the last decade, suggesting an accelerating pace of discovery and scholarly attention.

From January 2000 to August 2023, the number of published papers exhibited an exponential growth trend that can be modeled by the following equation: y = 2.0215^e0.2786x^, where y is the annual publication number and x is the number of years starting from 2000. R^^2^ = 0.9656, indicating an excellent fit. This trend is illustrated in Figure 2.

### 3.2 The most productive countries

Globally, 52 countries have contributed to the publications on MI-related lncRNAs. Table 1 displays the top 10 most productive countries in this field. China is the most prolific country, accounting for 75.65% of the total publications (N = 1,019; Total Citations, TC = 24,620), followed by the United States (N = 144; 10.69%; TC = 11,210), Germany (N = 57; 4.23%; TC = 6,059), Iran (N = 34; 2.52%; TC = 345), and Italy (N = 23; 1.71%; TC = 1,506). The remaining countries collectively contributed to 5.2% of the total number of publications.

**TABLE 1:** Top 10 most productive countries on research related to myocardial infarction-related lncRNAs.

SCR	Country	Articles	Percent (%)	TC	AAC	SCP	MCP	MCP_Ratio
1	China	1,019	75.65	24,620	24.20	961	58	0.057
2	United states	144	10.69	11,210	77.80	96	48	0.333
3	Germany	57	4.23	6,059	106.30	21	36	0.632
4	Iran	34	2.52	345	10.10	29	5	0.147
5	Italy	23	1.71	1,506	65.50	13	10	0.435
6	Canada	19	1.41	978	51.50	13	6	0.316
7	United kingdom	17	1.26	616	36.20	9	8	0.471
8	Egypt	14	1.04	116	8.30	10	4	0.286
9	France	11	0.82	605	55.00	4	7	0.636
10	Spain	9	0.67	254	28.20	7	2	0.222

SCR, standard competition ranking; TC, total citations; AAC, average article citations; SCP, single country publications; MCP, multiple country publications.

**FIGURE 3:**
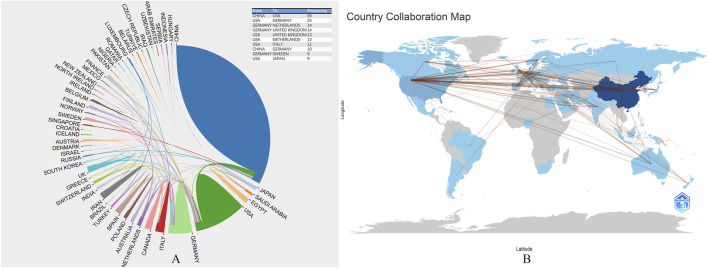
**(A)**, The collaborative research between countries and national literature published in the last 23 years; **(B)**, The map of global scientific collaboration. Each country is color-coded based on the number of publications originating from that region, with darker shades indicating higher publication counts. The map highlights key contributing countries demonstrating the global nature of research efforts in this field. Additional notes provide information on the sources of geographic data, the methodology for categorizing and visualizing publication counts, and any notable trends or patterns observed in the distribution of research activities.

Figures 3A, B show the collaboration network among all contributing countries. The international collaborations are primarily distributed across Asia, North America, and Europe. China is the most active country in collaborating with other nations, particularly with the United States (frequency = 56), followed by collaborations between the United States and Germany (frequency = 24), Germany and the Netherlands (frequency = 14).

### 3.3 The most productive institutions and cooperation

A total of 1,448 institutions have contributed to research on MI-related lncRNAs. Table 2 summarizes the top 10 institutions with the highest number of publications. Among the top 10, six are located in China, two in the United States, one in Egypt, and one in Germany. The top three most productive institutions are Harbin Medical University (N = 144), Harvard University (N = 118), and Southern Medical University-China (N = 68).

**TABLE 2:** Top 10 publishing volume organizations.

Affiliation	Articles	Country	Percentage (%)
Harbin Medical University	144	China	9.92
Harvard University	118	United states	8.13
Southern Medical University - China	68	China	4.68
Central South University	67	China	4.61
University of California System	45	United states	3.10
Chinese Academy of Medical Sciences - Peking Union Medical College	43	China	2.96
Zhengzhou University	43	China	2.96
Egyptian knowledge bank (EKB)	42	Egypt	2.89
German Centre for Cardiovascular Research	40	Germany	2.75
Sun Yat Sen University	40	China	2.75

Co-authorship analysis among different institutions was conducted to evaluate the underlying collaborative relationships. As shown in Supplementary Figure S1A, each institution had at least 10 papers, and the node size represents the number of papers, while the color signifies different communities. The network visualization includes 46 institutions, covering six communities, and represents stable scientific collaboration between the institutions. The total link strength was maximized at 256.

The institutional analysis in MI-related lncRNA research revealed evolving collaborations over time, as shown in Supplementary Figure S1B. The color indicates the average year of publication in specific research areas. Blue marks signify institutions that began publishing earlier, while shades from green to yellow represent more recently initiated institutional publishing. Research in this specific area was first initiated by the team from the University of California System between 2000–2007, followed by Harbin Medical University starting their research in 2007.

### 3.4 Journal contributions and citation analysis

A total of 1,452 papers on MI-related lncRNAs have been published in 445 journals. Table 3 shows the top 10 journals with a total of 273 publications, accounting for 19% of all publications. According to the Journal Citation Reports (JCR) categories, the top 10 journals are distributed as follows: Q1 (30%), Q2 (40%), and Q3 (30%). Most of the top 10 journals are based in the United States (N = 7), followed by one each in Italy, the United Kingdom, and Greece. The journal with the highest number of publications is the “European Review for Medical and Pharmacological Sciences” (N = 46). The number of articles published by the top 10 journals ranges from 19 to 46. “Circulation Research” (IF = 20.1) is the journal with the highest impact factor and the most citations (TC = 4,537).

**TABLE 3:** Top 10 most productive journals on research related to myocardial infarction-related lncRNAs.

SCR	Journals	Articles	Country	Total citations	IF (2022)	JCR
1	European Review for Medical and Pharmacological Sciences	46	Italy	1,051	3.3	Q3
2	Molecular Medicine Reports	33	Greece	515	3.4	Q3
3	Journal of Cardiovascular Pharmacology	29	United states	265	3	Q3
4	Arteriosclerosis Thrombosis and Vascular Biology	28	United states	2,532	8.7	Q1
5	Journal of Cellular Physiology	27	United states	723	5.6	Q2
6	Journal of Cellular Biochemistry	26	United states	513	4	Q2
7	Circulation Research	24	United states	4,537	20.1	Q1
8	Scientific Reports	22	England	802	4.6	Q2
9	Aging-US	19	United states	438	5.2	Q2
10	Bioengineered	19	United states	93	4.9	Q1

SCR, standard competition ranking; IF, impact factor; JCR, journal citation reports.

**FIGURE 4:**
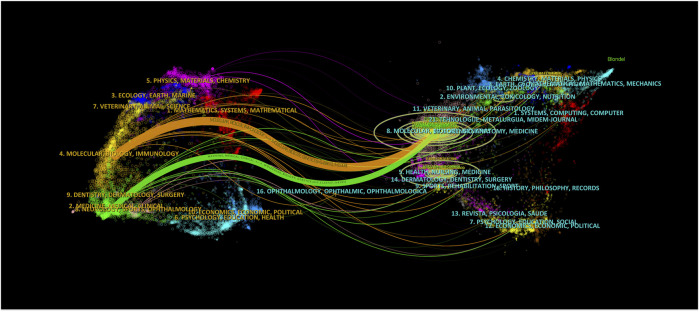
The knowledge flow in the field of MI-related lncRNAs in the last 23 years at the journal level. The overlay map is constructed based on journal citation data, visually representing the interdisciplinary connections and citation dynamics within the scientific community. The color coding of the nodes signifies different journal categories, such as cardiovascular research, molecular biology, and genetics. Edges between nodes indicate citation relationships, where the thickness of the edges reflects the frequency of citations between journals.

Supplementary Figure S2 displays the co-citation relationships between journals, based on a minimum co-citation count of at least 100. The network visualization map includes 128 journals covering four communities. The largest node is “Circulation Research,” with a total link strength of 964,366. Knowledge flow in the field of MI-related lncRNAs at the journal level can be obtained using the biplot overlay analysis function of CiteSpace 6.2.4.R4, applied to the journal data set covering the past 23 years. The progress of knowledge flow in the field over the past 23 years is shown in Figure 4. Journals in the categories of Molecular Biology and Immunology predominantly cite journals in the Molecular Biology and Genetics categories. The higher the z-value in the citation path, the more significant the proportion of applied citations. The maximum z-value in the graph is approximately 6.34.

### 3.5 Science overlay maps and interdisciplinary connections

**FIGURE 5:**
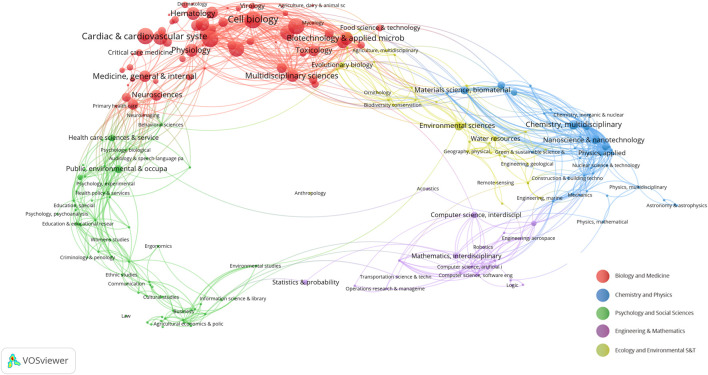
Overlay maps on the basis of a global map of science based on aggregated journal citation data. The nodes in the graph represent different scientific fields. Red: biology and medicine, blue: chemistry and physics, green: psychology and social sciences, purple: engineering and mathematics, yellow: ecology and environmental S&T. The connections between the nodes represent the connections between the fields.

We employ the methodology presented in “Science overlay maps: a new tool for research policy and library management” (Leydesdorff and Rafols, 2009; Rafols and Meyer, 2010) to construct overlay maps based on a global map of science using aggregated journal citation data from 2015. As illustrated in Figure 5, the research domains are primarily concentrated in the biological and medical areas, specifically in cardiovascular systems, cell biology, and physiology. Interestingly, there are also cross-disciplinary associations and influences with chemistry, applied physics, computer science, and statistics. This may imply a level of similarity and complementarity in both the content and methodology across these fields, indicating a potential for innovation.

### 3.6 The most influential authors and co-cited author analysis

A total of 7,181 authors have contributed to the field of MI-related lncRNAs. Table 4 showcases the top 10 most prolific authors, detailing their number of publications, H-index, G-index, M-index, and total citation counts. The most productive author is ZHANG Y from Harbin Medical University in China, with 43 publications (H-index = 18, TC = 2,166). He is closely followed by ZHANG L, with 32 publications (H-index = 20, TC = 1,237) and LI Y with 32 publications (H-index = 16, TC = 828). Supplementary Figure S3A delineates the collaborative networks among authors. The minimum criterion to be included in the network is at least 5 published papers. In this visual network map, 71 authors are represented, spanning 10 different communities. The largest node is Lu Yanjie, with a total link strength of 368, connected with the communities of Zhang Ying and Yang Fan. Temporal overlap in co-authorship tendencies is illustrated in Supplementary Figure S3B. The representation effectively captures the dynamic nature of collaborations, indicating potential strongholds of expertise and areas for future synergistic research endeavors.

**TABLE 4:** Top 10 most productive authors on research related to myocardial infarction-related lncRNA.

SCR	Author	NP	h_index	g_index	m_index	TC	PY_start
1	Zhang Y	43	18	43	1.059	2,166	2007
2	Zhang L	32	20	32	2	1,237	2014
3	Li Y	32	16	28	1.231	828	2011
4	Wang Y	31	13	24	1.444	591	2015
5	Wang Q	26	15	26	1.364	1,113	2013
6	Zhang J	25	12	25	0.571	908	2003
7	Liu Y	24	13	24	0.929	1,293	2010
8	Li J	21	12	21	0.857	1,299	2010
9	Wang J	21	11	21	1.571	525	2017
10	Li L	21	10	20	1.429	423	2017

SCR, standard competition ranking; NP, number of publications; TC, total citation; PY_start, the starting time of the publication year.

### 3.7 Research hotspots

#### 3.7.1 Most cited articles

Analysis of the most-cited papers provides valuable insights into pivotal contributions and impactful work in the specific research domain. Supplementary Table S1 lists the top 10 most-cited articles in the field of MI-related lncRNAs, each with over 700 citations and published between 2010 and 2018. The globally most-cited article is by Yap et al. (2010), with a global citation count (GC) of 1,063. The article sheds light on how Polycomb CBX7 in Polycomb Repressive Complex 1 binds to LncRNA-ANRIL to control cellular senescence. The locally most-cited article is by Vausort et al. (2014), with a local citation count (LC) of 127. The study measured the expression levels of five different lncRNAs in peripheral blood from 414 acute myocardial infarction (MI) patients who underwent direct percutaneous coronary intervention. The research found that levels of lncRNAs in blood cells are regulated after MI and may help in predicting outcomes, thus encouraging further investigation into the role of lncRNAs post-MI. In the list of top 10 most-cited articles in the field of MI-related lncRNAs, popular lncRNAs include MIAT, ANRIL, LincRNA-p21, LIPCAR, APF, and CAIF among others.

#### 3.7.2 Most frequent keywords

**FIGURE 6:**
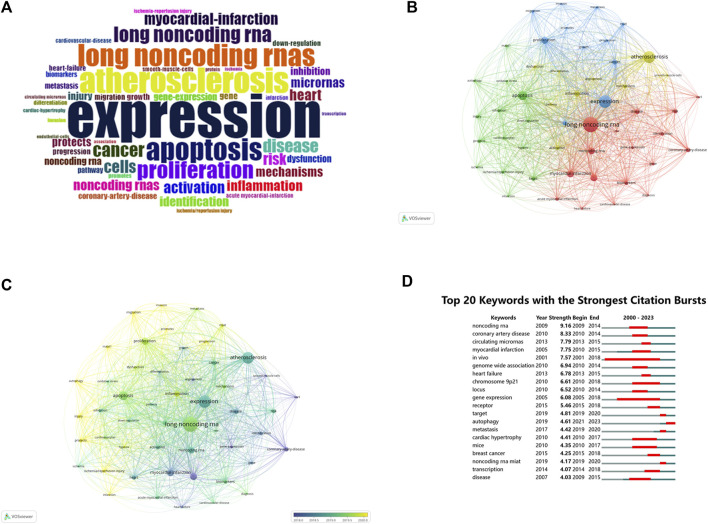
**(A)** Keyword wordcloud: Top 50 keywords in MI-related lncRNAs in the last 23 years. **(B)** Network visualization map of keyword co-occurrence on MI-related lncRNAs. **(C)** Network visualization map of the current tendency towards MI-related lncRNAs based on keywords analysis. **(D)** Top 20 keywords with the strongest citations bursts. The size of each node represents the frequency of a keyword, while the link between nodes indicates the relationship between nodes, with the distance between them indicating the strength of the connection. The color indicates the co-occurrence of keywords **(B)**, while the color change indicates the time change of keywords **(C)**. “Begin” and “End” refer to the start and end of the keyword emergence, respectively. Strength is a measure of the intensity of the cited change. Red or blue bars represent time intervals. Red bars indicate bursts of citations **(D)**.

Keyword analysis can reveal hotspots and trends within a specific research domain. Keyword analysis of the literature in this dataset was conducted using different software to analyze various research directions and hot topics. A total of 2,803 keywords were extracted from 1,452 articles. Figure 6A displays a word cloud of the 50 most frequent keywords in the field of MI-related lncRNAs. The top 10 most frequent keywords, all appearing more than 100 times, are as follows: “long noncoding rna” (N = 585), “expression” (N = 461), “atherosclerosis” (N = 422), “apoptosis” (N = 311), “myocardial infarction” (N = 227), “microrna” (N = 219), “proliferation” (N = 218), “noncoding rna” (N = 205), “inflammation” (N = 172), “cancer” (N = 149).

#### 3.7.3 Keyword co-occurrence and clustering

Based on a minimum co-occurrence count of 43, a stable network of the 51 most frequent keywords was visualized, composed of four clusters as shown in Figure 6B. The size of each node represents the frequency of each keyword, and the links between them signify their relationships.Cluster 1 (Red): Centered on cardiovascular diseases and gene expression, with keywords like “long noncoding rna,” “noncoding rna,” and “myocardial infarction.”Cluster 2 (Green): Focused on cardiac ischemia/reperfusion injuries and protection, featuring keywords such as “apoptosis,” “autophagy,” and “ischemia/reperfusion injury.”Cluster 3 (Blue): Concentrates on expression and proliferation, with popular keywords like “expression,” “proliferation,” and “cancer.”Cluster 4 (Yellow): Centers on atherosclerosis and inflammatory responses, featuring keywords such as “atherosclerosis,” “activation,” and “inflammation.” As shown in Figure 6C, the hotspots in this research area have evolved over time. Purple represents keywords widely used before 2018, while yellow represents those extensively used after 2020, with “long noncoding rna” dominating.

Supplementary Figure S4 shows the relationships among authors, institutions, and keywords in this field. The research team from Harbin Medical University led by Zhang Y frequently collaborates with Wang Q’s team from Southern Medical University, with mutual focus keywords being “atherosclerosis,” “long noncoding rna,” and “myocardial infarction.”

#### 3.7.4 Keyword burst analysis

The phenomenon when a keyword experiences a sudden surge in citations within a particular period is termed as a “keyword burst.” Figure 6D illustrates the top 20 keyword bursts in this dataset, with red lines indicating the period of bursts. In the first decade (2000–2010), the top five bursts were for “noncoding rna,” “coronary artery disease,” “myocardial infarction,” “*in vivo*,” and “genome-wide association.” In the most recent decade (2013-August 2023), the top five bursts are for “circulating micrornas,” “heart failure,” “receptor,” “target,” and “autophagy.” This burst analysis further elucidates hotspots and the evolutionary trajectory of research in the field of MI-related lncRNAs. The research focus has evolved from initial studies on genome-wide associations to more in-depth explorations into mechanisms like circulating micrornas, heart failure, and autophagy.

**FIGURE 7:**
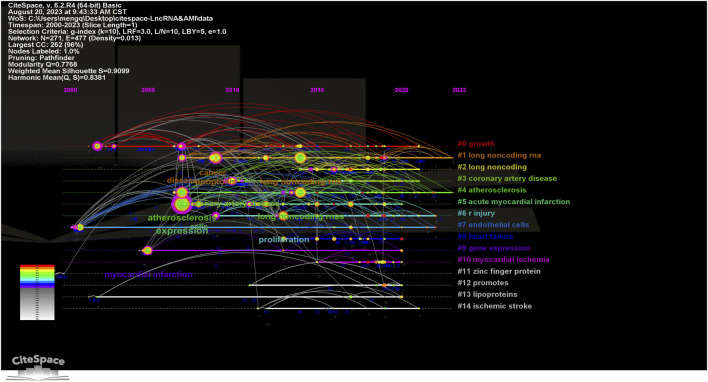
Timeline visualization map of keyword clustering in the field of MI-related lncRNAs research. The size of the nodes represents the number of citations of the word, the position of the nodes represents the point in time when it was noted, the thickness of the lines between the nodes represents the strength of the correlation, and the color represents the clustering of research hotspots.

A cluster analysis conducted on a data network built using CiteSpace 6.2.R4 further excavates key themes in this domain. A visual map of the keyword cluster timeline over the past two decades in the field of MI-related lncRNAs is displayed in Figure 7. The network comprises 271 nodes and 477 edges. The modularity (Q = 0.7768) being greater than 0.3 indicates significant clustering structures, and silhouette (S = 0.9099) further substantiates 15 highly significant thematic clusters, each represented along their respective timelines.

## 4 Discussion

To the best of our knowledge, this is the first comprehensive bibliometric analysis of literature concerning long non-coding RNAs (LncRNAs) associated with myocardial infarction (MI). This analysis aimed to assess research development over the past 23 years concerning MI-related lncRNAs. The literature from January 2000 to August 2023 has exhibited a continuous increasing trend, with an exponential growth pattern especially since 2018, publishing on average about 177.7 articles per year. This suggests increasing attention and interest in the potential role of lncRNAs in myocardial infarction, likely influenced by the high prevalence of cardiovascular diseases (Author Anonymous, 2021) and the pivotal role lncRNAs play in myocardial infarction (Ounzain et al., 2015; Zhang et al., 2016).

Secondly, China stands as the most productive country in the field, contributing 75.65% of the total publications. Although the United States and Germany produce fewer publications, their higher citation numbers suggest greater impact. Iran and Italy have also made notable contributions. International collaborations, especially between China and the United States, play a significant role, possibly due to varying levels of academic research support. Such international cooperation fosters interdisciplinary research and advances the field. Institutions like Harbin Medical University, Harvard University, and the University of California system have excelled in this research, indicating a stable scientific collaboration between institutions.

Additionally, an analysis of the publication years by institutions reveals that some organizations initiated their research at an early stage, while others have only recently begun to participate. Research on MI-related lncRNAs is chiefly disseminated across 445 journals within the realms of biology and medicine, with the top 10 journals accounting for 19% of the total publications. Circulation Research stands as the journal with the highest impact factor, underscoring the significance attributed to this area of study. The scope of this research encompasses multiple aspects including cardiovascular systems, cell biology, and physiology. Moreover, there is an interdisciplinary overlap with fields such as chemistry, applied physics, computer science, and statistics, highlighting the researchers’ quest to understand and harness the potential of MI-related lncRNAs across multiple disciplines. Such interdisciplinary research may offer novel perspectives and methodologies for the treatment and diagnosis of myocardial infarction.

Furthermore, key influential authors and hot keywords have been analyzed to identify renowned researchers, institutions, and key themes. For example, YAP KL’s work (Yap et al., 2010) on the interaction between Polycomb CBX7 and LncRNA-ANRIL has high citation counts, while VAUSORT M’s paper (Vausort et al., 2014) on peripheral blood lncRNA expression in acute myocardial infarction patients holds promise for prognostic applications. Research involving lncRNAs like MIAT, ANRIL, LincRNA-p21, and LIPCAR, among others, have gained considerable attention, revealing their diverse roles in vascular smooth muscle cell proliferation, apoptosis, and atherosclerosis. Authors such as Zhang et al. (2016), ZHANG L and LI Y have secured a prominent position in the research domain, as evidenced by their extensive publication records and high impact factors.

After myocardial infarction, the expression of lncRNAs changes significantly. Studies have shown that specific lncRNAs, such as ZFAS1 and CDR1AS, exhibit complementary changes following acute myocardial infarction, suggesting that these lncRNAs play important regulatory roles in cardiomyocyte injury and repair (Zhang et al., 2016). The expression levels of these lncRNAs can serve as biomarkers for myocardial infarction, aiding in the prediction of disease progression and patient prognosis. Several pivotal lncRNAs have garnered attention from researchers, such as MIAT, ANRIL, LincRNA-p21, LIPCAR, APF, CAIF. MIAT was identified in a large-scale case-control study (Ishii et al., 2006), and is a myocardial infarction susceptibility locus found on chromosome 22q12.1. MIAT regulates endothelial cell proliferation and apoptosis by acting as a ceRNA for miR-150, affecting VEGF expression and angiogenesis. MIAT also modulates inflammatory responses and oxidative stress, crucial in MI pathogenesis (Yan et al., 2015). ANRIL is located in the 9p21 genomic region, which also encodes cell cycle regulatory factors such as p14 (ARF), p15 (INK4b), and p16 (INK4a), potentially increasing the risk for clinical/subclinical coronary artery disease and aortic disease (Holdt et al., 2010). Further research suggests that ANRIL may modulate vascular smooth muscle cell proliferation, cell adhesion, and apoptosis through multiple mechanisms including cis, trans, and Polycomb complex protein binding, possibly contributing to the risk of coronary artery disease (Kotake et al., 2011; Pasmant et al., 2011; Congrains et al., 2012a; Congrains et al., 2012b; Motterle et al., 2012; Tsai et al., 2012; Ahmed et al., 2013; Bochenek et al., 2013; Holdt et al., 2013; Johnson et al., 2013; Chen et al., 2014). ANRIL interacts with PRC1 and PRC2 to repress CDKN2A/B, promoting VSMC proliferation and inhibiting apoptosis (Yap et al., 2010). ANRIL also regulates inflammatory gene expression and cellular senescence, contributing to atherosclerosis and CAD (Holdt et al., 2010; Congrains et al., 2012a; Congrains et al., 2012b). LincRNA-p21 enhances p53 activity to regulate the repression of target genes, promoting apoptosis of damaged cells and inhibiting vascular smooth muscle cell proliferation in response to ischemic injury (Huarte et al., 2010; Wu et al., 2014). LIPCAR acts as a biomarker for cardiac remodeling and heart failure post-MI by regulating mitochondrial function and apoptosis, influencing mitochondrial energy production and apoptotic pathways (Kumarswamy et al., 2014). APF promotes autophagy by targeting miR-188-3p, regulating autophagy-related genes, and improving cardiomyocyte survival during ischemic stress (Wang et al., 2015). CAIF inhibits autophagy by suppressing p53-mediated myocardin transcription, reducing excessive cellular component degradation during the acute phase of MI(28). Other lncRNAs involved in MI pathology: H19 regulates VSMC proliferation and apoptosis by acting as a ceRNA for miR-138 and miR-200a, influencing cell growth and apoptosis in atherosclerosis (Li et al., 2009; Gao et al., 2015). Linc00323-003 and MIR503HG modulate endothelial cell function and angiogenesis by regulating VEGF signaling, contributing to vascular homeostasis and repair (Fiedler et al., 2015). HIF1A-AS1 regulates apoptosis and proliferation of vascular endothelial cells by modulating the HIF1A signaling pathway, crucial for the response to hypoxia and ischemia (Wang et al., 2015). MALAT1 influences endothelial cell function and vessel growth by regulating cell cycle genes and alternative splicing of pre-mRNAs (Tripathi et al., 2013; Michalik et al., 2014). Lnc-Ang362 regulates VSMC proliferation and migration by modulating Ang II receptor expression and downstream signaling pathways, critical for vascular remodeling and hypertension (Leung et al., 2013). LncRNA-SENCR regulates endothelial cell migration and proliferation by modulating genes involved in VSMC differentiation and function (Bell et al., 2014). LncLSTR regulates lipid metabolism and homeostasis by modulating genes involved in lipid biosynthesis and transport, impacting lipid levels and atherosclerosis risk (Li et al., 2015). LncRNA RP5-833A20.1 modulates cholesterol homeostasis and inflammatory responses by acting as a ceRNA for miR-382-5p, influencing cholesterol metabolism and inflammatory signaling pathways (Hu et al., 2015). sONE regulates endothelial nitric oxide synthase (eNOS) expression and activity by acting as an antisense RNA, modulating NO production and vascular tone, critical for endothelial function and cardiovascular health (Robb et al., 2004; Fish et al., 2007). Although lncRNAs are generally unstable in cytoplasm (Kumarswamy et al., 2014) and susceptible to degradation, some display structural variations like folding and circularization, and can serve as biomarkers for myocardial infarction (Ounzain et al., 2015; Cai et al., 2016; Yan et al., 2016; Zhang et al., 2016).

Furthermore, keyword analysis reveals the primary research directions in this field, encompassing areas such as lncRNAs expression (Wang et al., 2016), cardiac ischemia/reperfusion injury (Liu et al., 2014; Liu et al., 2015), cell proliferation, and atherosclerosis. Through emerging keyword citation analysis, it is evident that hot topics in this area have evolved over different periods. From early gene association studies (Zangrando et al., 2014; Ounzain et al., 2015) to recent investigations into heart failure (Li et al., 2013; Yang et al., 2014; Di Salvo et al., 2015) and autophagy mechanisms (Wu et al., 2014; Zhang et al., 2019), the field of MI-related lncRNAs has been continually evolving. Autophagy is a critical cytoprotective mechanism during myocardial infarction. Studies have shown that certain lncRNAs, such as APF (Autophagy Promoting Factor) and CAIF (Cardiac Autophagy Inhibitory Factor), play significant roles in regulating autophagy-related gene expression, thereby influencing cardiomyocyte survival and apoptosis. APF promotes autophagy by targeting miR-188-3p (Wang et al., 2015), while CAIF inhibits autophagy by suppressing p53-mediated myocardin transcription (Liu et al., 2018). These findings provide potential targets for developing new therapeutic strategies for myocardial infarction. These research trends may reflect the developmental trajectory and focal points of future studies in this domain. In the context of myocardial infarction and LncRNAs, the frequent appearance of the terms “expression,” “atherosclerosis,” and “apoptosis” highlights their interconnected roles in cardiovascular pathology. “Expression,” lncRNAs regulate gene expression through various mechanisms, including chromatin remodeling, transcriptional regulation, RNA processing, and post-transcriptional regulation. Specifically, MIAT has been identified as a myocardial infarction susceptibility locus in a large-scale case-control study, and is also implicated in diabetic retinopathy, microvascular dysfunction, and neuronal development (Ishii et al., 2006). Additionally, ANRIL interacts with multiple proteins, such as those in the Polycomb complex, to regulate gene expression and influence vascular smooth muscle cell proliferation and atherosclerosis progression (Holdt et al., 2016). Atherosclerosis is a major cause of myocardial infarction, and lncRNAs play a significant role in its development. For example, ANRIL regulates vascular smooth muscle cell proliferation, adhesion, and apoptosis, increasing the risk of coronary artery disease and arterial disease (Holdt et al., 2010). lncRNA H19 is associated with atherosclerosis risk, with polymorphisms in H19 linked to this condition in a Chinese population (Gao et al., 2015). “Apoptosis,” or programmed cell death, lncRNAs are crucial in regulating apoptosis in cardiomyocytes, which is essential for cell survival post-myocardial infarction. For instance, lncRNA ZFAS1 induces mitochondrial-mediated apoptosis by causing cytosolic Ca2^+^ overload (Jiao et al., 2019). Understanding these relationships underscores the significance of LncRNAs in myocardial infarction, offering insights into their potential as focal points for novel therapeutic strategies.

The COVID-19 pandemic has significantly impacted cardiovascular health globally, with evidence suggesting that SARS-CoV-2 infection can lead to myocardial injury and inflammatory responses (Bangalore et al., 2020; Clerkin et al., 2020; Guo et al., 2020; Shi et al., 2020; Zhou et al., 2020). Emerging research indicates that long non-coding RNAs (lncRNAs) may play a crucial role in these processes. For instance, COVID-19 infection has been shown to differentially regulate several lncRNAs, affecting their normal functions and potentially exacerbating cardiovascular complications. The infection induces a robust inflammatory response, often leading to a cytokine storm, which can impact the expression and function of these lncRNAs. For example, MALAT1 is downregulated, possibly diminishing its protective roles. NEAT1 is upregulated, potentially exacerbating inflammation and myocardial damage. These alterations in lncRNA expression and function highlight the complex interplay between viral infection and host cellular mechanisms, underscoring the importance of understanding these interactions for better management of cardiovascular complications in COVID-19 patients (Greco et al., 2020). Moving forward, it is expected that research in this area will focus on understanding the mechanisms by which lncRNAs influence myocardial inflammation and repair in the context of COVID-19, as well as the long-term cardiovascular effects of the virus.

Lifestyle factors such as physical activity, nutrition, and sleep have been shown to significantly affect cardiovascular health and the incidence of MI. Studies have demonstrated that regular physical activity can improve cardiovascular outcomes and reduce the risk of MI (You et al., 2024a). Nutrition, particularly diets rich in fruits, vegetables, and omega-3 fatty acids, has been linked to reduced inflammation and improved cardiovascular health (You et al., 2024b). Additionally, adequate sleep is crucial for maintaining cardiovascular health, with sleep disturbances being associated with an increased risk of MI (You et al., 2024c). While the direct mechanisms linking lifestyle factors to lncRNA expression in the context of MI remain unclear, it is plausible that these factors could influence lncRNA-mediated pathways indirectly through their overall impact on cardiovascular health. Future research should explore these potential interactions to provide a more comprehensive understanding of how lifestyle modifications can be leveraged to prevent and manage MI through lncRNA regulation.

Limitations of this study include its reliance on the WoSCC database (Donthu et al., 2021), which, while comprehensive, may not include all relevant studies. The majority of publications being in English could also introduce selection bias. This study limits its literature review to the period from 2000 to 2023, potentially omitting valuable insights from earlier foundational research.

In summary, significant progress has been made in the field of MI-related lncRNAs over the past few years; however, numerous enigmas and challenges still remain. The sustained growth and international collaboration in this research area will aid in deepening our understanding of the role of lncRNAs in myocardial infarction, and offer more possibilities for the development of new diagnostic methods and therapeutic strategies.

This study serves as a comprehensive guide for emerging scholars in the domain of MI-related lncRNA research. It delineates the evolution of the field, encapsulating historical developments, the current research landscape, and prospective avenues of investigation. By synthesizing these elements, the research furnishes scholars with a robust framework to navigate the intricacies of the field, enabling them to formulate pertinent research inquiries and contribute effectively to the scholarly discourse.

## 5 Conclusion

Over the past two decades, the field of MI-related lncRNAs has experienced significant growth, showing an exponential trend. China holds considerable influence in this domain, with collaborations spanning across Asia, North America, and Europe. The research is concentrated in areas including cardiovascular systems, cell biology, and physiology. Our bibliometric analysis reveals critical biological insights into the roles of lncRNAs in MI, particularly their regulatory functions in gene expression, atherosclerosis, and apoptosis. These lncRNAs not only serve as potential biomarkers but also offer promising therapeutic targets for MI. Future studies may likely focus on areas such as circulating microRNAs, heart failure, receptors, targets, and autophagy, which hold high research potential. This bibliometric analysis on MI-related lncRNAs offers a comprehensive perspective on the current status and future trends in this field, providing a framework for integrating bibliometric tools with biological research to advance our understanding and treatment of MI.
