# Supplementary material for: Progress and trends in myocardial infarction-related long non-coding RNAs: a bibliometric analysis
Source: Front Mol Biosci. 2024 Jul 29;11:1382772. doi: 10.3389/fmolb.2024.1382772 (PMC11317247; doi:10.3389/fmolb.2024.1382772)
Supplement: Supplementary file 1 [file DataSheet1.DOCX]

Supplementary Material

# Supplementary Table

Table S1. Top 10 most cited articles on Myocardial Infarction-Related LncRNAs

| **SCR** | **Author & Year** | **Title** | **Journal** | **TC** | **Normalized Citations** |
| --- | --- | --- | --- | --- | --- |
| 1 | VAUSORT M, 2014 | Long noncoding RNAs in patients with acute myocardial infarction | CIRC RES | 127 | 7.56 |
| 2 | YAN BA, 2015 | lncRNA-MIAT regulates microvascular dysfunction by functioning as a competing endogenous RNA | CIRC RES | 101 | 13.56 |
| 3 | HOLDT LM, 2010 | ANRIL Expression Is Associated With Atherosclerosis Risk at Chromosome 9p21 | ARTERIOSCL THROM VAS | 81 | 4.05 |
| 4 | WU GZ, 2014 | LincRNA-p21 regulates neointima formation, vascular smooth muscle cell proliferation, apoptosis, and atherosclerosis by enhancing p53 activity | CIRCULATION | 81 | 4.82 |
| 5 | KUMARSWAMY R, 2014 | Circulating long noncoding RNA, LIPCAR, predicts survival in patients with heart failure | CIRC RES | 73 | 4.35 |
| 6 | YAP KL, 2010 | Molecular interplay of the noncoding RNA ANRIL and methylated histone H3 lysine 27 by polycomb CBX7 in transcriptional silencing of INK4a | MOL CELL | 54 | 2.70 |
| 7 | WANG K, 2015 | APF lncRNA regulates autophagy and myocardial infarction by targeting miR-188-3p | NAT COMMUN | 53 | 7.11 |
| 8 | QU XF, 2017 | MIAT is a pro-fibrotic long non-coding RNA governing cardiac fibrosis in post-infarct myocardium | SCI REP-UK | 49 | 6.23 |
| 9 | CONGRAINS A, 2012 | Genetic variants at the 9p21 locus contribute to atherosclerosis through modulation of ANRIL and CDKN2A/B | ATHEROSCLEROSIS | 48 | 6.48 |
| 10 | LIU CY, 2018 | LncRNA CAIF inhibits autophagy and attenuates myocardial infarction by blocking p53-mediated myocardin transcription | NAT COMMUN | 47 | 8.69 |

Notes: TC: Total local citation; Normalized Citations: The number of local citations of the article divided by the average number of citations of all articles in local field.

# Supplementary Figures

## Figure S1A. Network visualization map of institution co-authorship analysis on Myocardial Infarction-Related LncRNAs

## Figure S1B. Network visualization map for the current tendency towards Myocardial Infarction-Related LncRNAs based on institution co-authorship analysis

## Figure S2. Network visualization map of journals on Myocardial Infarction-Related LncRNAs based on co-authorship analysis

## Figure S3A. Network visualization map of co-authorship analysis on Myocardial Infarction-Related LncRNAs

## Figure S3B. Network visualization map for the current tendency towards Myocardial Infarction-Related LncRNAs based on co-authorship analysis

## Figure S4. Three-Fields Plot of the keyword analysis (Author-Institution-Keyword)

## Supplementary Figures


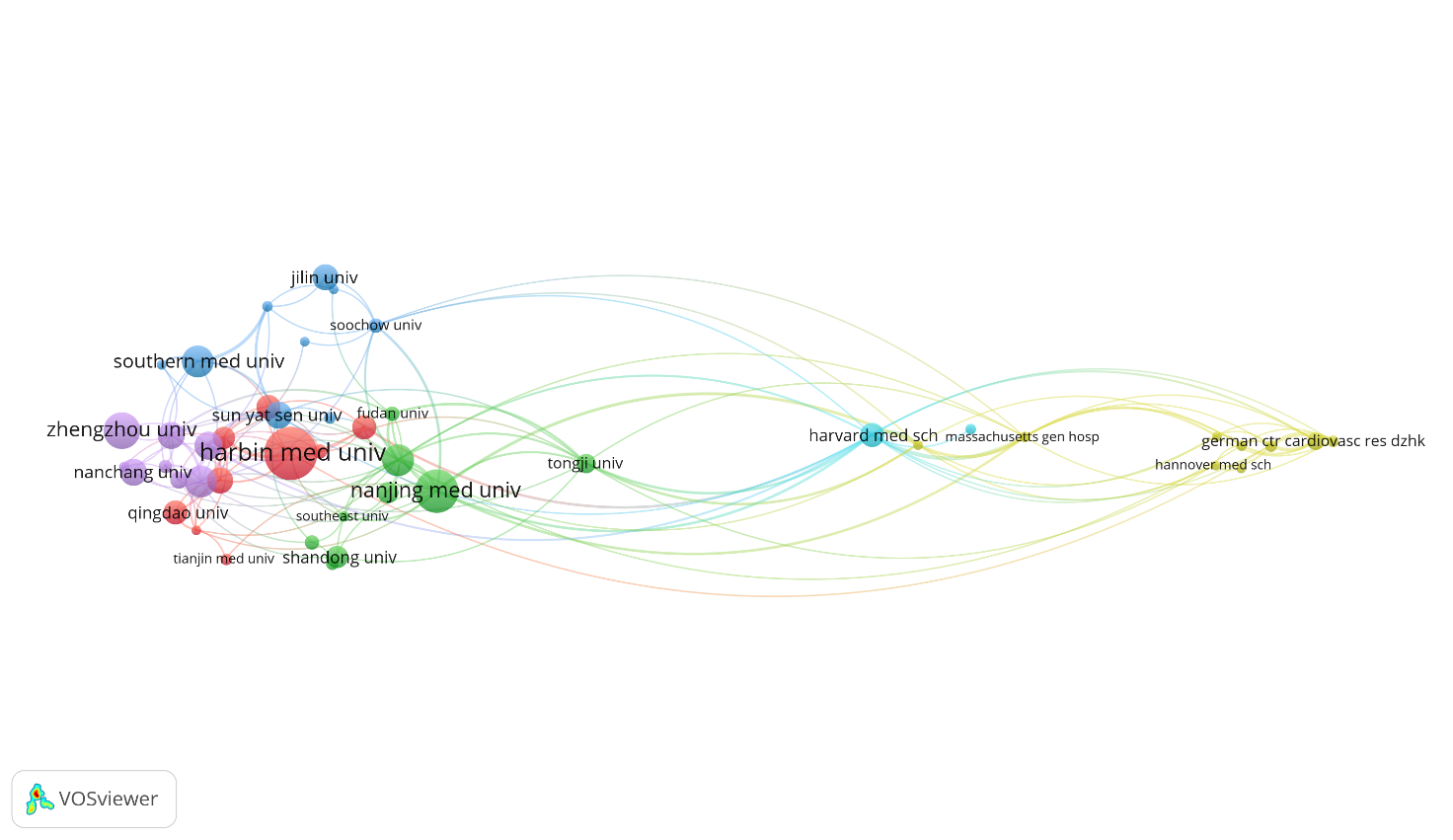


**Supplementary Figure S1A.** Network visualization map of institution co-authorship analysis on Myocardial Infarction-Related LncRNAs


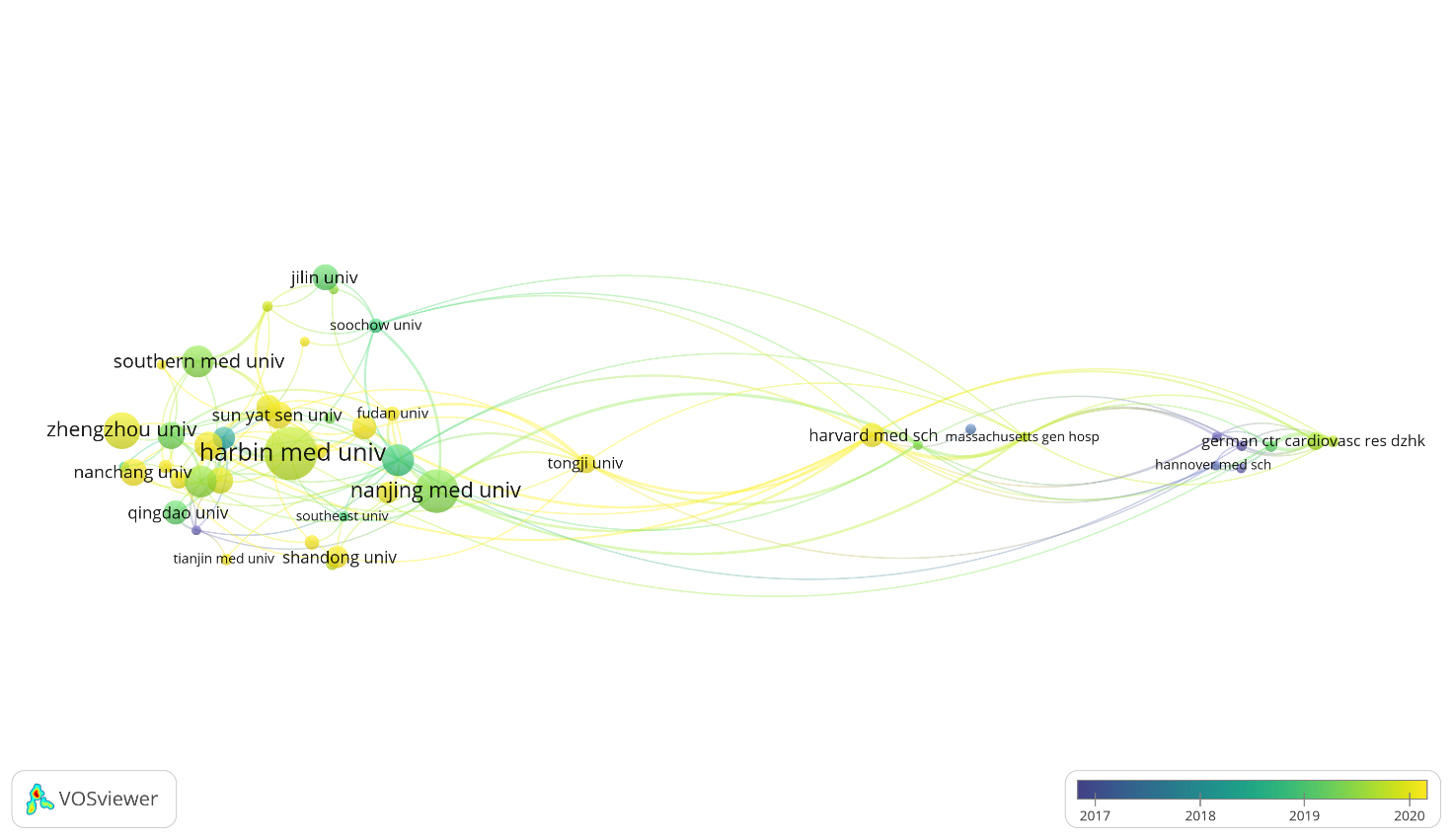


**Supplementary Figure** **S1B.** Network visualization map for the current tendency towards Myocardial Infarction-Related LncRNAs based on institution co-authorship analysis

Notes: The size of each node represents the number of articles, with a minimum number of 10 publications in each institution; the link between nodes indicates the relationship between nodes, with the distance between them indicating the strength of the connection. The color indicates different communities (Figure S1A), while the color change indicates the time change of institution’s publications (Figure S1B).


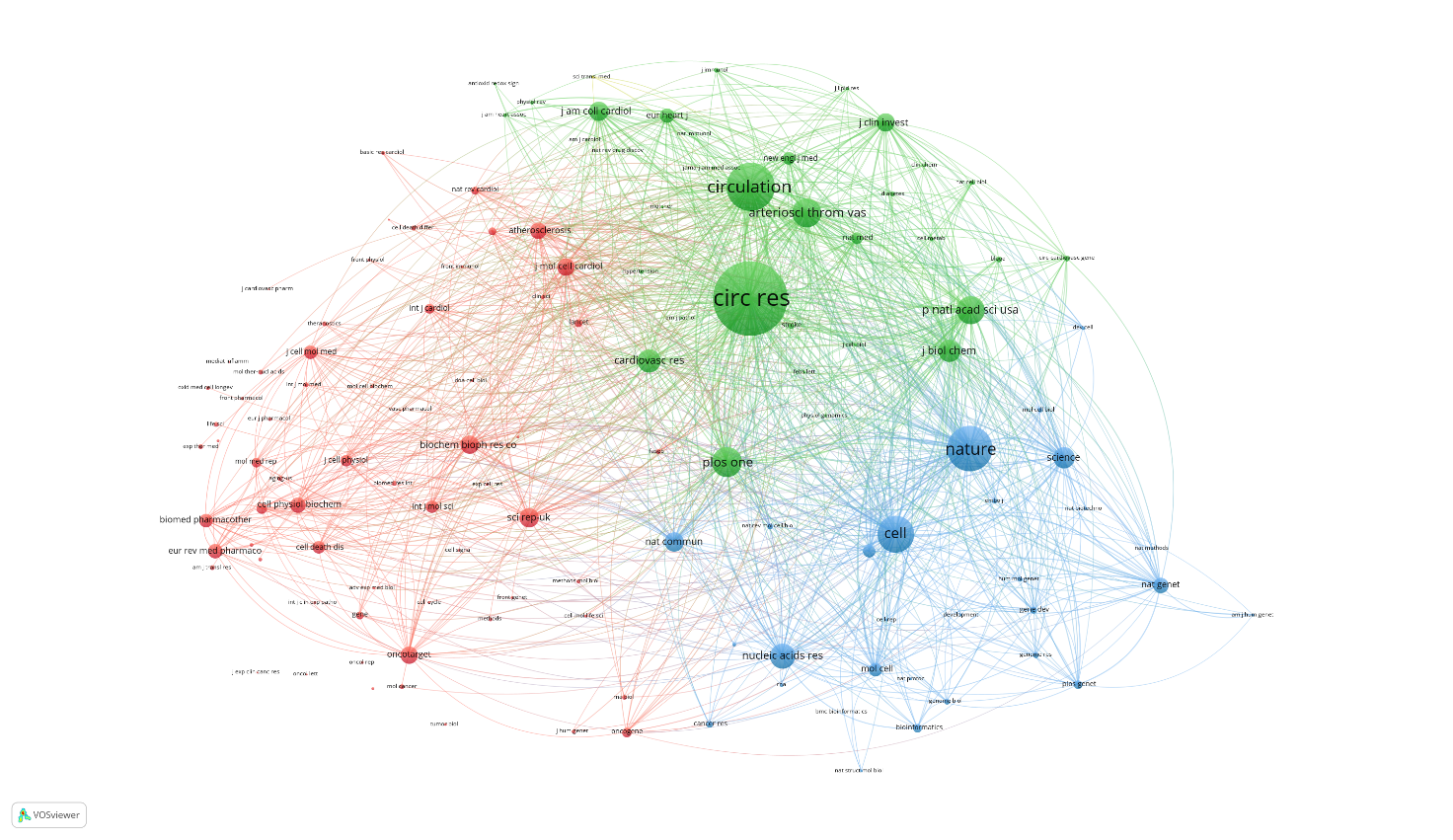


**Supplementary Figure S2.** Network visualization map of journals co-authorship analysis on Myocardial Infarction-Related LncRNAs

Notes: The size of each node represents the number of articles, with a minimum number of 100 co-cited publications in each journal; the link between nodes indicates the relationship between nodes, with the distance between them indicating the strength of the connection. The color indicates different communities.


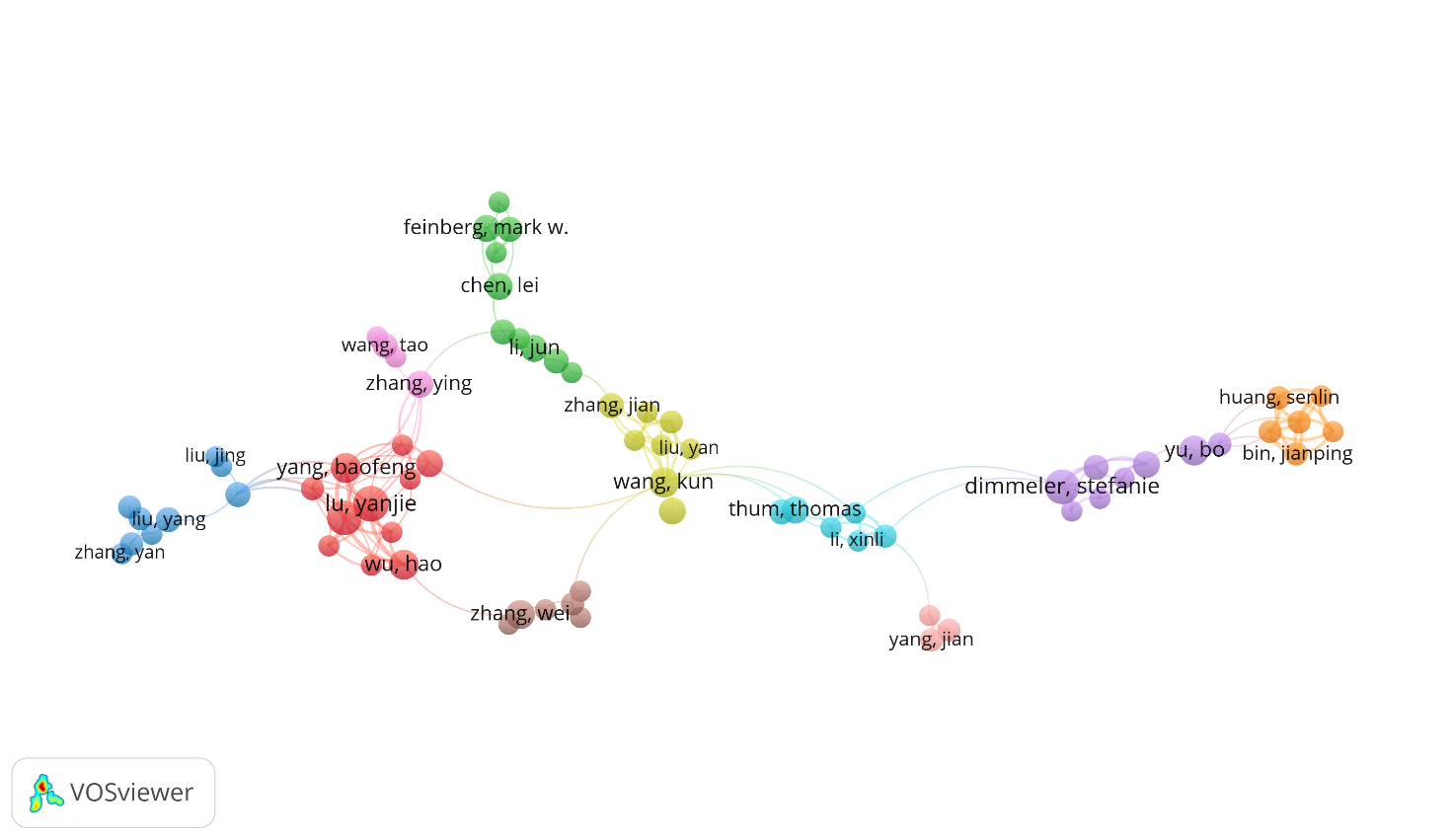


**Supplementary Figure S3A.** Network visualization map of co-authorship analysis on Myocardial Infarction-Related LncRNAs


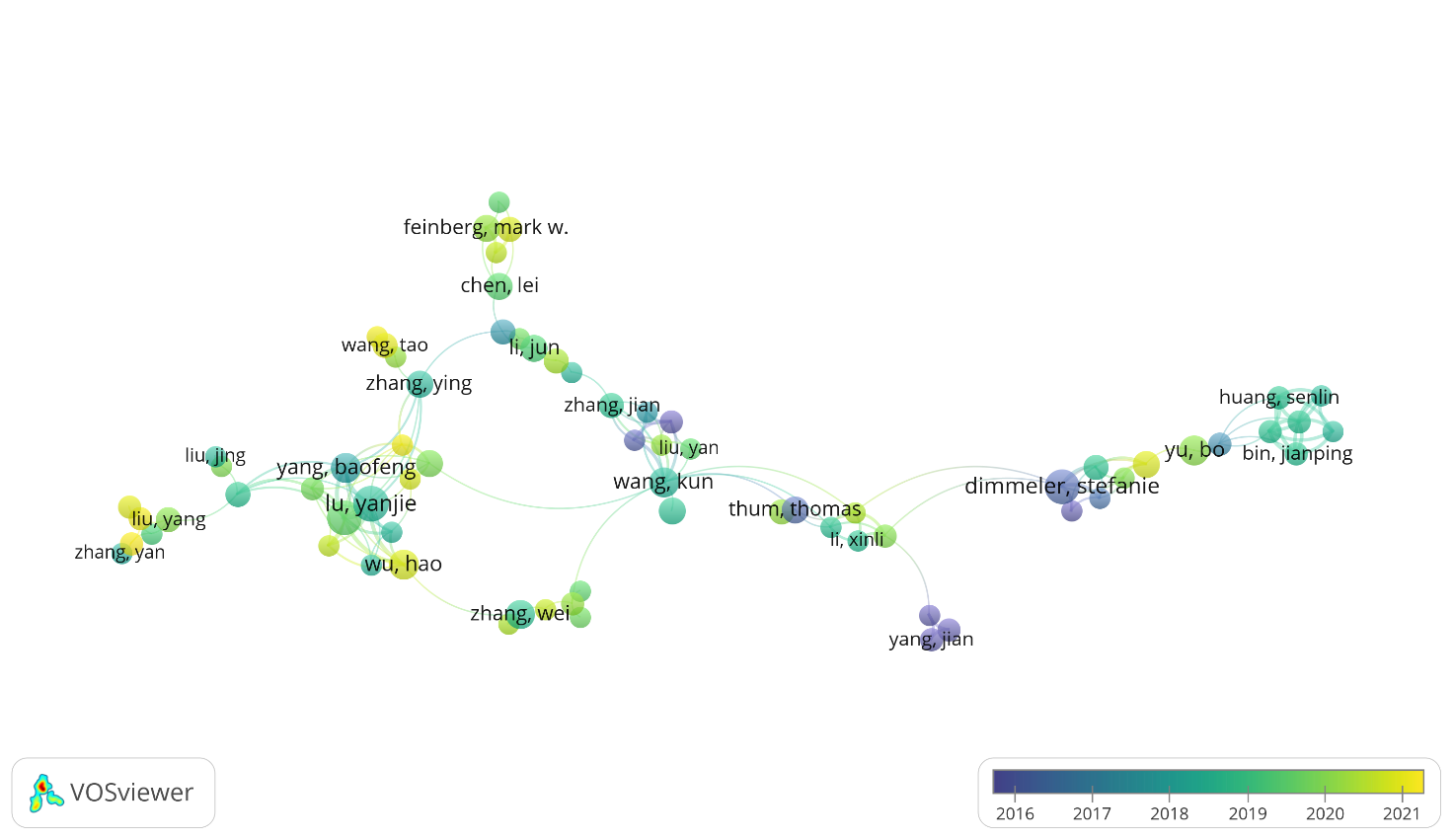


**Supplementary Figure S3B.** Network visualization map for the current tendency towards Myocardial Infarction-Related LncRNAs based on co-authorship analysis

Notes: The size of each node represents the strength of the respective institution co-authorship; the link between nodes indicates the relationship between nodes, with the distance between them indicating the strength of the connection. The color indicates the co-occurrence of institution (Figure S3A), while the color change indicates the time change of institution co-authorship (Figure S3B).

**
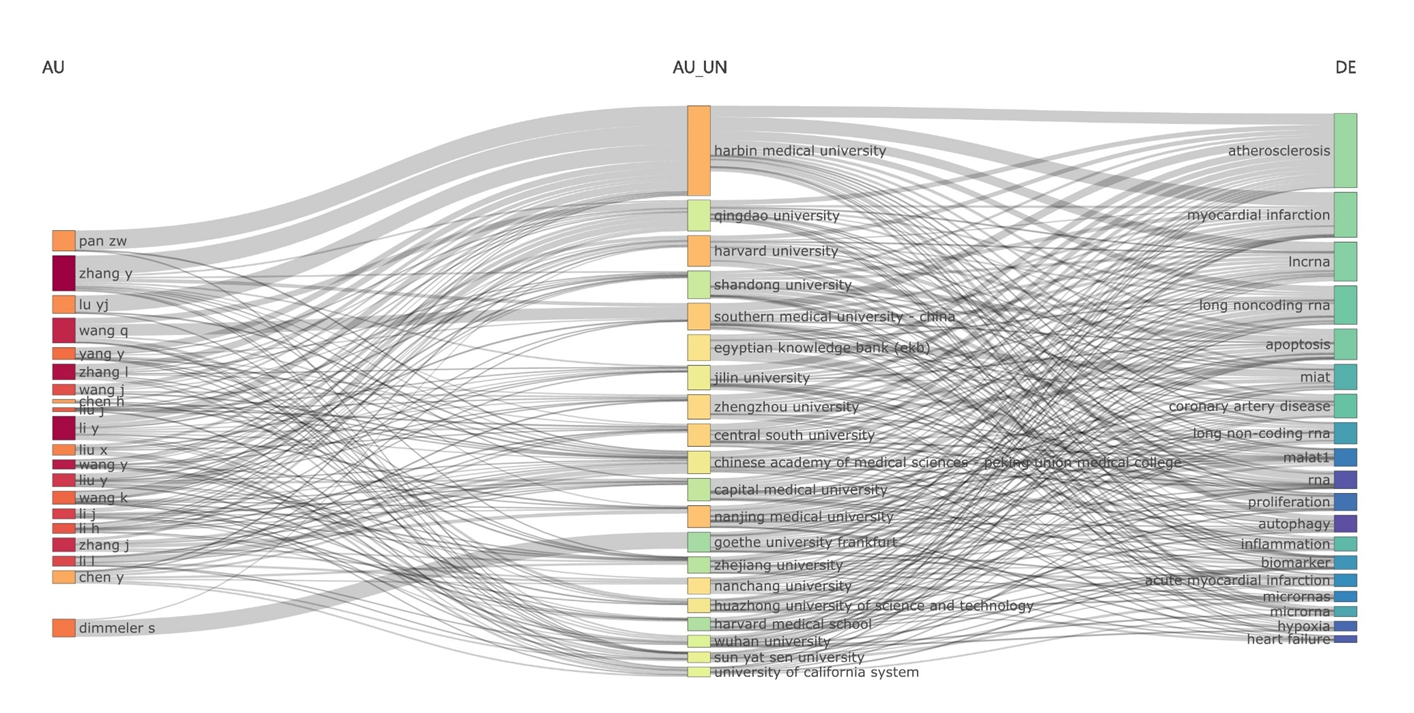
**

**Supplementary Figure** **S4.** Three-Fields Plot of the keyword analysis (Author-Institution-Keyword)

Notes: Three-field plot of the keywords analysis: (left field: authors; middle field: affiliations; right field: keywords)
